# Advances in the treatment of secondary and tertiary hyperparathyroidism

**DOI:** 10.3389/fendo.2022.1059828

**Published:** 2022-12-06

**Authors:** Li-Xi Zhang, Ben Zhang, Xu-Yao Liu, Zi-Ming Wang, Peng Qi, Tong-Yue Zhang, Qiang Zhang

**Affiliations:** Thyroid Surgery Department, General Surgery Center, First Hospital of Jilin University, Changchun, China

**Keywords:** secondary hyperparathyroidism, tertiary hyperparathyroidism, parathyroid hormone, 99mTc-MIBI, parathyroidectomy

## Abstract

Secondary hyperparathyroidism (SHPT) and tertiary hyperparathyroidism (THPT) are common and complicated clinical endocrine diseases. The parathyroid glands maintain endocrine homeostasis by secreting parathyroid hormone to regulate blood calcium levels. However, structural alterations to multiple organs and systems occur throughout the body due to hyperactivity disorder in SHPT and THPT. This not only decreases the patients’ quality of life, but also affects mortality. Since current treatments for these diseases remains unclear, we aimed to develop a comprehensive review of advances in the treatment of SHPT and THPT according to the latest relevant researches.

## 1 Introduction

The parathyroid gland is a group of glands located behind thyroid, usually four (1). Hyperparathyroidism (HPT) is characterized by abnormal calcium and phosphorus metabolism caused by excessive secretion of parathyroid hormone (PTH). This is mainly classified as primary, secondary, and tertiary hyperparathyroidism. Primary hyperparathyroidism (PHPT) refers to parathyroid gland hyperfunction caused by hyperplasia, neoplasia, or malignancy. Secondary hyperparathyroidism (SHPT) is a type of hypermetabolism caused by other diseases, like chronic renal failure (2), post-hemodialysis (3), kidney transplantation (4), and bariatric surgery (5) and leads to increased blood calcium from increased PTH reacting to low blood calcium levels. In tertiary hyperparathyroidism (THPT), long-term overstimulation of the parathyroids leads to the development of nodules due to the compensatory hyperfunction to spontaneous PTH incretion. THPT is common in patients with long-term chronic kidney disease, hemodialysis, and kidney transplantation (6). HPT can cause bone diseases (7), such as pathological fractures or skeletal deformities, cardiovascular diseases (8), and kidney stones and may also induce end-stage parathyroid cancer, although this is extremely rare (9, 10). Serum intact parathyroid hormone (iPTH) measurement and technetium-99m sestamibi (99mTc-MIBI) parathyroid imaging are the most effective and reliable methods for diagnosis. Diagnostic sensitivity is further improved by adding ultrasonography (11). HPT is complex, diverse, and seriously endangers patients’ quality of life. However, there is currently no unified treatment, especially regarding the choice of surgical interventions and clinical drug therapeutics. This review introduces comprehensive advances in SHPT and THPT treatment based on our own practical experience and the latest national and international reports. We provide a reference for clinicians to make individualized treatment decisions and to benefit more patients.

## 2 Treatment for HPT

### 2.1 Medical therapy for SHPT and THPT

Academics generally believe that THPT is not amenable to medication, due to the nodules that autonomously secrete PTH. Once the diagnosis of THPT has been verified, surgery is recommended as long as there are no contraindications. Therefore, we will concentrate on medical treatment for SHPT.

#### 2.1.1 Phosphorus binders

SHPT is often associated with hyperphosphatemia; thus, phosphate binders are often used. Phosphate binders maintain normal serum calcium and PTH levels by correcting hyperphosphatemia. There are calcium-containing (e.g., calcium carbonate) and non-calcium-containing (e.g., sevelamer) phosphorus binders.

Calcium carbonate inhibits the secretion of PTH and is often used with vitamin D analogs; however, hypercalcemia can easily occur (12).

Sevelamer is a primary drug of choice for SHPT (13). It rapidly reduces blood phosphorus concentration, inhibiting parathyroid cell proliferation and reducing PTH levels (14). However, its severe gastrointestinal side effects, such as vomiting and abdominal pain, greatly reduce patient compliance (13).

A novel oral non-calcium-containing phosphorus binder, PA21, has recently emerged. PA21 is composed of polynuclear iron-hydroxide, sucrose, and starch. It effectively relieves hyperphosphatemia (15) and significantly decreases PTH levels. It is extremely effective for SHPT (16), and will likely become the drug of choice for SHPT associated with hyperphosphatemia in the future.

#### 2.1.2 Vitamin D and analogs

Vitamin D deficiency can lead to insufficient calcium absorption and result in excessive PTH production, causing SHPT. Thus, vitamin D and its analogs treat SHPT by correcting the associated vitamin D deficiency (17). Commonly used medications include calcitriol, paricalcitol, and alfacalcidol.

Calcitriol promotes intestinal calcium absorption (18) and also improves bone metabolism by inhibiting osteoclasts and promoting osteoblasts. Both oral and intravenous administration can be used to treat SHPT (19). Calcitriol is more effective than alfacalcidol in lowering serum PTH (20), but often induces hypercalcemia and hyperphosphatemia (21).

Paricalcitol is a selective vitamin D receptor activator that significantly reduces PTH secretion without affecting calcium and phosphorus levels (22), i.e., it has a favorable safety profile. Even if occasional hypercalcemia and hyperphosphatemia occur (21), the incidence of these side effects is less than with calcitriol (22). Thus, it may be the best vitamin D analog for SHPT in the future.

DP001(2MD) is a novel oral selective vitamin D analog with high selectivity for bone and the parathyroid glands. It binds to vitamin D receptors and inhibits PTH synthesis and secretion. It is safer than existing active vitamin D analogs because it is rapidly and widely distributed to the target tissues and has a long half-life (21). Therefore, it is likely to have broader application in the future.

#### 2.1.3 Calcimimetics

Calcium-sensing receptors in the parathyroid glands are important therapeutic targets for SHPT. Therefore, calcimimetics may be administered, but not with phosphorus binders and vitamin D analogs, to reduce PTH secretion by activating calcium-sensing receptors, thus effectively controlling calcium and phosphorus levels (23). Cinacalcet is the most common calcimimetic.

Cinacalcet is an allosteric activator of calcium-sensing receptors. It increases the sensitivity of calcium-sensing receptors to extracellular calcium, and allosterically combines with the receptors to inhibit PTH secretion (24). It is effective in infants and young children (25), as well as THPT patients who cannot undergo parathyroid surgery (26). Long-term use of cinacalcet reduces total parathyroid volume (27). Serum PTH, calcium, and alkaline phosphatase increase significantly within 12 months of cinacalcet discontinuation (28); therefore, continuous use is recommended. Cinacalcet may induce gastrointestinal symptoms, as well as drug-drug interactions, which interfere with patient compliance (29, 30). Hypocalcemia occurs in some patients, but it is generally mild or asymptomatic and resolves spontaneously (31). Calcium-sensing receptor polymorphisms (32) or the presence of gallstones (33) can interfere with cinacalcet’s efficacy. Recent studies have found that combined use of cinacalcet and vitamin D significantly reduces serum calcium and phosphorus levels without increasing side effects, which offers a favorable option for the SHPT treatment (34).

Etelcalcetide is a synthetic peptide that has been approved in several countries as the only intravenous calcimimetic for SHPT treatment. It consistently and effectively reduces PTH, calcium, and phosphorus levels (30). Etelcalcetide may slow the progression of SHPT by reducing levels of fibroblast growth factor 23 (FGF-23), a hormone that regulates phosphate and vitamin D metabolism (35). When FGF-23 is elevated, parathyroid cell proliferation is induced and PTH secretion is accelerated, leading to refractory SHPT (36). Intravenous administration avoids liver metabolism (37) and reduces gastrointestinal adverse reactions. Thus, it is safe and well-tolerated, with improved patient compliance (38). However, etelcalcetide may still cause side effects, the most common being hypocalcemia (39, 40) (**Table 1**). Because etelcalcetide is similar to cinacalcet in terms of safety, efficacy, and side effects (23)(**Table 1**), if patient compliance is poor, it is recommended that etelcalcetide administered intravenously three times a week should be used instead of daily oral cinacalcet.

**Table 1:** Comparison of treatments.

Comparison of treatments	Efficacy in patients with SHPT			Adverse events	References
	Follow-up time	Target	Response rate		
Etelcalcetide vs. Cinacalcet	26 weeks	Reduction in PTH of more than 30%	68.2% vs. 57.7%	Decreased blood calcium (68.9% vs. 59.8%)	(40)
	9 months	PTH level of 150–600 pg/mL	48-62% vs. 40-47%		(23)
Evocalcet vs. Cinacalcet	30 weeks	PTH level of 60-240 pg/mL	72.7% vs. 76.7%	Gastrointestinal-related adverse events (18.6% vs. 32.8%)	(29)
sPTX vs. tPTX-AT	6 months	PTH level	270.6 pg/mL vs. 320.9 pg/mL	Recurrent HPT (5.6% vs. 4%) Persistent HPT (21.6% vs. 16%)	(41)
	6 months	PTH level of 15-65 pg/mL	22.2% vs. 26.7%	Recurrent or persistent HPT (26% vs. 8.7%)	(42)
sPTX vs. tPTX-45AT vs. tPTX-90AT	12 months	PTH level of 100–600 pg/mL	44% vs. 33% vs. 45%	Un-controlled HPT (20% vs. 0% vs. 0%)	(43)

Considering the disadvantages of cinacalcet and etelcalcetide in terms of dosage and side effects, a new-generation oral calcium-sensing receptor modulator, evocalcet, has been studied and marketed (29). It is not inferior to cinacalcet in inhibiting iPTH, and also prevents parathyroid hyperplasia (44). It has fewer gastrointestinal-related side effects (29) (**Table 1**), and is therefore a promising prospect and is expected to be put into clinical use as soon as possible.

#### 2.1.4 Other medical treatments

In addition to phosphorus binders, vitamin D and its analogs, and calcimimetic agents, other SHPT treatment options include bisphosphonates (drugs inhibiting bone resorption) (7), synbiotics (combinations of prebiotics and probiotics) (45), and denosumab (monoclonal antibody) (46). Denosumab especially is used in patients with inoperable THPT (46). However, these treatments are not widely used in clinical practice due to their unknown mechanisms of action or unknown safety owing to a lack of repeated studies.

### 2.2 Surgical treatment of SHPT and THPT

Surgery is the best treatment option for patients who have failed medical therapy or those with advanced SHPT and THPT. At present, the main surgical methods are parathyroidectomy (PTX) and ablation.

Preoperative localization diagnosis is important for HPT surgery, especially for recurrent or persistent HPT. Clinically, even in the SHPT and THPT ultrasound evaluation, ultrasound is the most commonly and necessary used means of evaluation of previously undiagnosed thyroid nodules, resulting in thyroidectomy at the same time of parathyroidectomy (47). Ultrasonography is easy to perform but has poor sensitivity. 99mTc-MIBI single-photon emission computed tomography associated with computed tomography scintigraphy (SPECT/CT) improves the sensitivity of preoperative parathyroid localization and accurately locates ectopic parathyroid glands (6). As a result, for refractory SHPT, ultrasound combined with SPECT/CT is the best choice for preoperative localization (48), while CT and MIBI scans are effective imaging modalities for the assessment of postoperative residual parathyroid glands and prior to repeat PTX (49). However, the exploration of the parathyroid glands by experienced surgeons is the key to successful HPT surgery.

#### 2.2.1 PTX

PTX effectively prevents persistent and recurrent HPT, and significantly reduces patient mortality (43). It is a safe and effective treatment for severely affected, frail, or pediatric patients (50).

##### 2.2.1.1 Surgical approach

The main surgical PTX procedures include total parathyroidectomy (tPTX), subtotal parathyroidectomy (sPTX), and total parathyroidectomy with autotransplantation (tPTX-AT). All surgical methods effectively control HPT, iPTH, calcium, phosphorus, and other biochemical parameters and clinical symptoms also improve (51).

tPTX involves the removal of all parathyroid glands and suspected parathyroid tissue. The risk of postoperative recurrence is low (52), but chronic hypoparathyroidism is more likely (53). This is not generally considered a first-line treatment.

sPTX retains normal vascular parathyroid tissue that is approximately 30-50 mg or two to three parathyroid glands in size (43). The advantage of this procedure is that the incidence of postoperative hypocalcemia is low; however, the residual parathyroid tissue is prone to recurrence (52). On this basis, some experts have proposed a “near total parathyroidectomy,” which leaves very small (3 mm × 3 mm × 3 mm) islands of parathyroid residues in situ. Postoperative follow-up found a low recurrence rate of HPT and normal parathyroid function (54). Since sPTX reduces the risk of hypoparathyroidism, it is generally preferred for THPT therapy (55).

tPTX-AT involves the removal of all parathyroid glands, and the normal parathyroid tissue (30-50 mg, is cut into 1-2 mm^3^ fragments and transplanted into the forearm muscle (mostly) or sternocleidomastoid muscle without arteriovenous fistula, and sometimes even into subcutaneous tissues. This procedure avoids postoperative hypocalcemia, is more effective at improving quality of life than PTX alone, and the iPTH level is not affected by the quantity and quality of graft fragments (43)(**Table 1**). However, a limitation of this procedure is that autologous parathyroid transplantation may lead to HPT recurrence (52), but the persistence and recurrence probability of HPT are much less than sPTX (41, 42)(**Table 1**). Thus, some experts recommend tPTX-AT as the preferred surgical management method for SHPT, because reoperation at the forearm autograft site is simpler than in the neck after sPTX (43). In addition to routine serum calcium, phosphorus, and alkaline phosphatase checks, blood samples should be collected from both forearms for the post-tPTX-AT evaluation of iPTH (43). In previous studies, the iPTH of the transplanted side was more than 1.5 times greater than that of the contralateral side, indicating graft viability, and the iPTH value of the contralateral side reflected the generalized iPTH level.

Some scholars have proposed the “subtotal parathyroidectomy and relocation of remnants,” which refers to the removal of all hyperplastic parathyroid glands and repositioning of a small portion (50 mg) of vascularized parathyroid tissue on the muscular surface of the subhyoid band, below the skin incision, which has a good supply of blood vessels. This not only preserves the ability of the residual glands to continue to secrete PTH and reduces the probability of postoperative complications, but also does not require re-exploration after recurrence, and thus, avoids damage to the recurrent laryngeal nerve (56).

In addition to parathyroid tissue, the hypothalamus and thymus also express PTH mRNA. During embryonic development, ectopic or supernumerary parathyroid glands may be formed (57), and the incidence of ectopic parathyroid glands in SHPT patients is 26%. In addition, the number of parathyroid glands is variable, with patients typically presenting with 3 to 8 parathyroid glands (1).At most ectopic parathyroid glands are found in the thymus, so thorough neck exploration is necessary to reduce the risk of postoperative recurrence and reoperation (58). To this end, a new surgical approach, namely decontamination PTX, has been proposed. This involves complete removal of the thyroid cartilage, bilateral carotid sheaths, the fibrofatty tissue around the innominate artery, and complete removal of the parathyroid glands. It has achieved a more durable and reliable effect in terms of controlling PTH levels (57).

The transoral vestibular (59) and three-port submental (60) endoscopic approaches commonly used in thyroidectomy are also safe and feasible for PTX, providing the best cosmetic results (59). Removal of all parathyroid glands (60), especially the ectopic parathyroid glands within the upper mediastinum (59), will most likely be popularized in the future. All parathyroid glands must be explored regardless of the surgical approach and the adipose tissue surrounding the glands must be completely removed (61).

##### 2.2.1.2 Intraoperative assistive technologies

Intraoperative assistive technologies are also required. Nanocarbon suspension-assistance with negative parathyroid imaging can protect the thyroid gland and recurrent laryngeal nerve (62), improve success rates, and reduce complications. Intraoperative neuromonitoring is also effective for patients receiving PTX (63). In recent years, near-infrared fluorescence imaging has received increasing attention due to its high tissue penetration and tissue autofluorescence (64). The parathyroid glands exhibit an 8.5-fold higher autofluorescence than do the surrounding tissues, including the thyroid (65). As an innovative point-of-care surgical imaging tool, it has a high sensitivity in detecting adenomas and parathyroid hyperplasia (65); however, this technique can only play a secondary role due to the low and uneven autofluorescence distribution in SHPT lesions (66). Indocyanine green is a water-soluble molecule that, when bound to proteins, emits strong fluorescence with a peak wavelength of about 830nm when excited by near-infrared fluorescence. This imaging has high sensitivity in distinguishing pathological parathyroid glands during surgery and can also predict postoperative hypocalcemia (67). Thus, imaging with indocyanine green during PTX helps surgeons to detect and verify the parathyroid glands (64). Intraoperative PTH detection not only contributes to the sensitivity and accuracy of predicting early treatment outcomes, but also reduces recurrence rates (68). Generally, iPTH levels at 20 minutes post-surgery are used to predict the PTX success rate. Compared with preoperative levels, an iPTH decrease rate >70% is the criterion for successful surgery (69). At this time, PTH has high sensitivity and specificity, and can reflect long-term levels (70).

##### 2.2.1.3 Postoperative-related complications and recurrence

Common complications after PTX include hungry bone syndrome, hypocalcemia, and hyperkalemia (71, 72). Age, preoperative alkaline phosphatase, calcium, iPTH values, and total weight of the removed parathyroid glands are all important risk factors for the development of hungry bone syndrome (73). Hungry bone syndrome or hypoparathyroidism can also cause severe postoperative hypocalcemia, manifested as muscle spasms (71). This is a major cause of readmission in patients after PTX (72). Therefore, close monitoring of postoperative serum calcium levels and intensive calcium supplementation are required in young patients with high preoperative alkaline phosphatase and PTH levels (74). Many patients develop hyperkalemia after PTX, and preoperative serum potassium is the only independent predictor (75). Therefore, regular and comprehensive assessments of serum biochemistry and timely correction are essential to reduce the incidence of complications.

Recurrence of HPT is also common after PTX, with iPTH levels >300pg/mL after 6 months (76). This is associated with hypertension, elevated creatinine, and elevated alkaline phosphatase (77). In combined forearm autograft surgery, it is recommended to extract PTH from the distal end of the graft site to avoid obtaining falsely high PTH levels, leading to a misdiagnosis of recurrent HPT (78). Reoperation is a safe and effective treatment for recurrence after PTX (79).

#### 2.2.2 Ablation

Ablation is a minimally invasive surgery mainly used for the treatment of tumors and heart-related diseases, etc. Different types of ablations include ethanol injection, microwave, radiofrequency, and laser. These techniques are also applicable for HPT patients. However, ablation is not often used clinically because incomplete ablation after ethanol injection leads to a high 1-year SHPT recurrence rate (around 80%) (80). Microwave ablation (MWA) and radiofrequency ablation (RFA) are favored for HPT patients with increased surgical risk because they are less invasive and have a shorter treatment time and faster postoperative recovery. Ablation is also feasible in hyperplasia of the four glands. In case of that, ablation of all four glands at one time is recommended to better control the iPTH value (80, 81).

MWA is performed by inserting an ablation needle into the parathyroid tissue under ultrasound guidance and is terminated when the entire nodule is hypoechoic and no flow signal is detected (82). MWA is safe for the treatment of HPT as it can destroy parathyroid tissue (83). It can also be used for the treatment of SHPT ectopic nodules (84), and all parathyroid glands found during surgery should be completely ablated. Attention should always be paid to the adjacent tissues and organs, such as the esophagus and recurrent laryngeal nerve, to avoid damage (85). However, MWA should not be used as an initial treatment for HPT, because most patients do not respond significantly, which reduces its effectiveness (50, 82). It is recommended for patients with poor health status that are unable to undergo PTX. Hypocalcemia may also occur after MWA (86).

RFA involves the complete ablation of hyperplastic parathyroid glands with a radiofrequency generator and cooling electrodes under ultrasound guidance (80). This can also be used to treat HPT. Like WMA, RFA is also prone to post-operative hypocalcemia, but the incidence is lower in patients who have undergone two RFAs (80). Although RFA is a safe method, similar to MWA, most patients are not sensitive to this treatment; thus, it has only limited usefulness in HPT patients (87).

### 2.3 Other potential treatments

In addition to the treatments described above, photodynamic therapy has recently been proposed. Originally developed for cancer, photodynamic therapy is also used in various clinical fields such as skin, venereal, and vascular diseases. Administration of 5-aminolevulinic acid (5-ALA) at specific sites leads to the accumulation of photosensitizing protoporphyrin-IX in the heme biosynthetic pathway. Irradiation with light intensity at an excitation wavelength excites porphyrin-IX and initiates a series of photochemical reactions responses, leading to cell damage and death (88, 89). Studies have found that after intraperitoneal injection of 5-ALA into rats, light irradiation of the parathyroid glands can destroy the parathyroid gland tissue to treat SHPT (88); thus, this also provides a new idea for the clinical treatment of SHPT.

## 3 Summary and outlook

While our understanding of all aspects of SHPT and THPT remains limited, treatment methods have been continuously developed and improved in recent years. These methods emphasize the importance of early detection, diagnosis, and treatment. In particular, surgeons continue to innovate with minimally invasive and open procedures, and propose new surgical techniques and approaches. This improves the success rate of interventions and reduces postoperative complications, as new treatment schemes for patients with related diseases are provided. However, because medications are expensive and the postoperative complication and mortality rates remain high, current treatment of SHPT and THPT still faces many challenges. In the future, doctors will need to continue to pay attention to these diseases, further explore effective drug development and treatment, rationally design surgical plans, improve surgical quality, and conduct early intervention and reasonable treatment for patients at risk. In this way, we can delay or prevent progression of the disease and improve patient quality of life as well as survival.

## Author contributions

This mini review is the result of the contributions of all authors. QZ and L-XZ conceived and designed the study. L-XZ and BZ wrote the manuscript. X-YL, Z-MW, PQ, and T-YZ reviewed and edited the manuscript. All authors contributed to the article and approved the submitted version.
